# Synthesis, Biological Evaluation, and Docking Studies of Novel Bisquaternary Aldoxime Reactivators on Acetylcholinesterase and Butyrylcholinesterase Inhibited by Paraoxon

**DOI:** 10.3390/molecules23051103

**Published:** 2018-05-07

**Authors:** Kamil Kuca, Daniel Jun, Lucie Junova, Kamil Musilek, Martina Hrabinova, Jorge Alberto Valle da Silva, Teodorico Castro Ramalho, Marian Valko, Qinghua Wu, Eugenie Nepovimova, Tanos Celmar Costa França

**Affiliations:** 1 Center for Basic and Applied Research, Faculty of Informatics and Management, University of Hradec Kralove, 50003 Hradec Králové, Czech Republic; teo@dqi.ufla.br (T.C.R.); tanos@ime.eb.br (T.C.C.F.); 2 Faculty of Military Health Sciences, University of Defence, Trebesska 1575, 50001 Hradec Kralove, Czech Republic; daniel.jun@unob.cz (D.J.); junova.lucie@gmail.com (L.J.); martina.hrabinova@unob.cz (M.H.); 3 Biomedical Research Center, University Hospital Hradec Kralove, Sokolska 581, 50005 Hradec Kralove, Czech Republic; kamil.musilek@gmail.com; 4 Faculty of Science, Department of Chemistry, University of Hradec Kralove, Rokitanskeho 62, 50003 Hradec Kralove, Czech Republic; capalberto05@yahoo.com.br (J.A.V.d.S.); wqh212@hotmail.com (Q.W.); Eugenie.nepovimova@uhk.cz (E.N.); 5 Laboratory of Molecular Modeling Applied to the Chemical and Biological Defense, Military Institute of Engineering, Praça General Tiburcio 80, Urca, 22290-270 Rio de Janeiro, RJ, Brazil; 6 Laboratory of Molecular Modeling, Chemistry Department, Federal University of Lavras, 37200-000 Lavras, MG, Brazil; 7 Faculty of Chemical and Food Technology, Slovak University of Technology, 81237 Bratislava, Slovakia; marian.valko@gmail.com; 8 College of Life Science, Yangtze University, Jingzhou, Hubei 434025, China

**Keywords:** acetylcholinesterase, antidote, butyrylcholinesterase, organophosphate, oxime, paraoxon

## Abstract

Nerve agents and oxon forms of organophosphorus pesticides act as strong irreversible inhibitors of two cholinesterases in the human body: acetylcholinesterase (AChE; EC 3.1.1.7) and butyrylcholinesterase (BChE; EC 3.1.1.8), and are therefore highly toxic compounds. For the recovery of inhibited AChE, antidotes from the group of pyridinium or bispyridinium aldoxime reactivators (pralidoxime, obidoxime, HI-6) are used in combination with anticholinergics and anticonvulsives. Therapeutic efficacy of reactivators (called “oximes”) depends on their chemical structure and also the type of organophosphorus inhibitor. Three novel oximes (K131, K142, K153) with an oxime group in position four of the pyridinium ring were designed and then tested for their potency to reactivate human (*Homo sapiens sapiens*) AChE (*Hss*ACHE) and BChE (*Hss*BChE) inhibited by the pesticide paraoxon (diethyl 4-nitrophenyl phosphate). According to the obtained results, none of the prepared oximes were able to satisfactorily reactivate paraoxon-inhibited cholinesterases. On the contrary, extraordinary activity of obidoxime in the case of paraoxon-inhibited *Hss*AChE reactivation was confirmed. Additional docking studies pointed to possible explanations for these results.

## 1. Introduction

Cholinesterase reactivators are generally used as a part of the antidotal therapy against pesticide and nerve agent poisonings [1]. They reactivate human acetylcholinesterase (*Hss*AChE) inhibited by organophosphorus (OP) compounds via breaking down the bond between inhibitor and enzyme [2]. Another possible use of *Hss*AChE reactivators is their utilization as human butyrilcholinesterase (*Hss*BChE) reactivators. This approach, called “pseudocatalytic scavenging”, is nowadays well-investigated as a possible prophylaxis against nerve agent poisonings [3,4].

**Figure 1:**
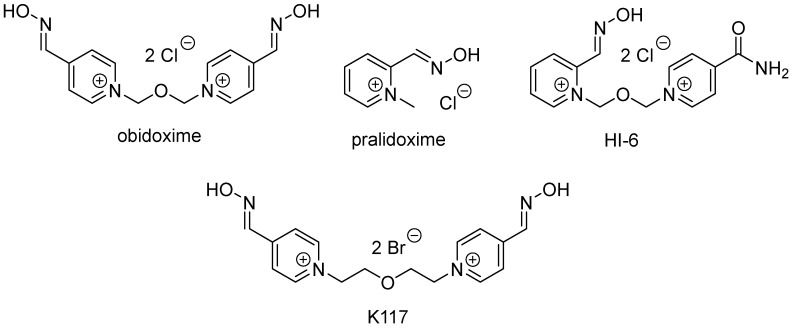
Chemical structures of clinically used AChE reactivators and oxime K117.

It is known that currently commercially available oximes are not able to satisfactorily reactivate *Hss*BChE inhibited by all pesticides or nerve agents [5]. This fact is caused by differences in structure between *Hss*AChE and *Hss*BChE [6]. Due to this, searching for oximes able to reactivate *Hss*BChE is needed. In our recent study, we have tested 23 structurally different oximes, which were originally developed for *Hss*AChE reactivation, as *Hss*BChE reactivators. As it resulted, the maximal reactivation activity was obtained for K117 (Figure 1) (16%), which is not enough if the pseudocatalytic scavenger application is considered [7].

Owing to these results, we wanted to test relatively new oximes and verify their potency to reactivate *Hss*AChE and *Hss*BChE inhibited by paraoxon (POX). The structures of the newly designed reactivators have the oxime group in position four on the pyridinium ring. This position is believed to be favorable if pesticide- and tabun-inhibited cholinesterases are reactivated [8,9]. Different heterocycles (pyridine, quinoline, and isoquinoline) were used as the second quaternary part of new reactivators. The choices of quinoline and isoquinoline as second quaternary parts was based on the fact that quaternary quinoline and isoquinoline compounds are strong inhibitors of *Hss*AChE and therefore have high affinity towards this enzyme [10,11].

In this study, all these three oximes were experimentally evaluated as reactivators of POX-inhibited *Hss*AChE and *Hss*BChE and compared with three standard commercially available oximes: pralidoxime (2-PAM), obidoxime, and HI-6 (Figure 1). Also, in order to investigate the interactions of the new oximes with the enzymes in comparison with the commercial one, and search for explanations for possible differences in the reactivation rates, docking studies were performed on the binding modes of all oximes inside the active sites of the complexes *Hss*AChE/POX and *Hss*BChE/POX, to evaluate the energies of their preferential poses adopted to achieve the appropriate angle and position for the attack to the OP bound to the enzyme, and enable the reactivation reaction.

## 2. Materials and Methods

### 2.1. Synthesis and Characterization of the New Oximes

**Figure 2:**
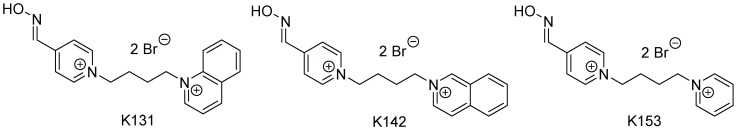
Chemical structures of the cholinesterase reactivators synthesized.

All the new oximes (Figure 2) were synthesized at our department using the standard synthetic approach described earlier [11,12]. Purity of these compounds was checked using TLC, HPLC, NMR, and elementary analysis immediately prior to the experiment [13,14].

### 2.2. Biochemical Experiments

POX was purchased from Dr. Ehrenstorfer (Augsburg, Germany) in 95% purity. Reactivation activity of synthesized reactivators was tested using our in vitro reactivation test [5,7,15]. A short description of this method is summarized here: erythrocyte *Hss*AChE or plasma *Hss*BChE was inhibited by solution of POX to 5% of their original activity. Time of enzyme inhibition with POX (2 hours, corresponding to 7 × T_1/2_) was calculated from the experimentally determined half-life (T_1/2_) of the reaction between enzyme and paraoxon. Then, inhibited enzyme was incubated for 10 min with solution of reactivator at concentration 10^−4^ and 10^−5^ M. Activity of *Hss*AChE (or *Hss*BChE) was measured spectrophotometrically by a modified method according to Ellman with acetylthiocholine (butyrylthiocholine) as substrate [16]. Reactivation potency was calculated from the equation: %R = [1 − (a_0_ − a_r_)/(a_0_ − a_i_)] × 100; where %R is the percentage of reactivation, a_0_ is the activity of intact enzyme, a_i_ is the activity of inhibited enzyme and a_r_ is the activity of reactivated enzyme minus oximolysis. Each measurement was repeated three times and was conducted under standard laboratory temperature (25 °C). Calculations were performed using the software GraphPad Prism version 4.00 for Windows, GraphPad Software, San Diego California USA (www.graphpad.com).

All experiments were carried out in compliance with the current law of the Czech Republic.

### 2.3. Molecular Modeling Studies

The 3D structures of 2-PAM, obidoxime, and HI-6 (Figure 1) and the oximes in Figure 2 were built using the program PC Spartan pro^®^ [17] and their partial atomic charges calculated by the AM1 semiempirical method [18]. Afterward, the most likely ionization form of each oxime under physiological pH (7.4) was determined using the web-based resource software Chemicalize, available at https://chemicalize.com/welcome, supported by ChemAxon Ltd [19].

The structures of *Hss*AChE and *Hss*BChE used in this work were obtained from the Protein Data Bank (PDB) (http://www.rcsb.org/pdb/home/home.do) under the codes 5HF9 and 4B0O, respectively. 5HF9 corresponds to the crystallographic structure of *Hss*AChE inhibited by POX and complexed with HI-6. This experimental structure was opened in the Discovery Studios^®^ Visualizer (DS^®^ Vis) 4.5 program [20], and had the missing loops PGGTGG and PKA and the C-terminal sequence EGR built through alignment to its FASTA sequence (code P22303) obtained from the Uniprot server (http://www.uniprot.org/), following the same procedure adopted before [21], in order to obtain a complete model for the complex *Hss*AChE/POX. 4B0O corresponds to the crystallographic structure of *Hss*BChE inhibited by soman and complexed with benzyl pyridinium-4-methyltrichloroacetimidate (BP4M). After being downloaded from the PDB, the soman structure was corrected to POX through the DS^®^ Vis 4.5 program [20] in order to generate the model of the complex *Hss*BChE/POX. Redocking studies of the respective crystallographic ligands (HI-6 and BP4M) were performed with both models in order to validate the docking protocol used.

The docking energies of the oximes inside the active sites of the complexes *Hss*AChE/POX and *Hss*BChE/POX, were obtained using the software Molegro Virtual Docker (MVD)^®^ [22] and the algorithm MolDock Score, an adaptation of the algorithm ”differential evolution” (DE) [22]. The binding site was restricted into a sphere with a radius of 15 Å, and the residues within a radius of 10 Å were considered flexible. Due to the stochastic nature of the docking algorithm, about 10 runs were performed for each compound with 100 poses (conformation and orientation of the ligand) returned to analysis. The best conformation of each oxime was selected according to the energy, the distance P_OP_ − O_Ser203_ (d_OP_), and the angle O_ox_ − P_OP_ − O_Ser203_ (θ_OPO_). For each oxime, we also computed the percentage of low-energy poses with distance d_OP_ < 10.00 Å and angle θ_OPO_ = 180 ± 60° (called the near attack conformations, or NAC) for comparison to the percentages of reactivation.

## 3. Results and Discussion

### 3.1. Characterization Data of the New Oximes

*4-hydroxyiminomethyl-1,1′-(but-1,4-diyl)-1-pyridinium-1′-quinolinium dibromide* (**K131**)*.* M.p. 177–179 °C. Yield 21%. ^1^H-NMR (300 MHz, DMSO-*d*_6_): δ (ppm) 9.68 (d, 1H, *J* = 5.8 Hz, H-2′), 9.34 (d, 1H, *J* = 8.4 Hz, H-8′), 9.12 (d, 2H, *J* = 6.2 Hz, H-2,6), 8.71 (d, 1H, *J* = 8.8 Hz, H-4′), 8.53 (d, 1H, *J* = 8.1 Hz, H-5′), 8.45 (s, 1H, -CH=NOH), 8.33–8.17 (m, 4H, H-3,3′,5,7′), 8.11-8.02 (m, 1H, H-6′), 5.17 (t, 2H, *J* = 7.0 Hz, N′-CH_2_-), 4.71 (t, 2H, *J* = 7.0 Hz, N-CH_2_-), 2.20–1.93 (m, 4H, N-CH_2_-CH_2_-). ^13^C-NMR (75 MHz, DMSO-*d*_6_): δ (ppm) 149.71, 148.29, 47.41, 145.01, 137.33, 135.58, 130.70, 129.81, 129.66, 123.99, 122.16, 119.00, 59.33, 58.35, 27.36, 25.88. EA: calculated 48.85 % C, 4.53% H, 8.99% N; found 48.41% C, 4.63% H, 9.06% N. ESI-MS: *m*/*z* 153.6 [M/2]^2+^ (calculated [C_19_H_21_N_3_O/2]^2+^ 153.59).

*4-hydroxyiminomethyl-1,1′-(but-1,4-diyl)-1-pyridinium-1′-isoquinolinium dibromide* (**K142**)*.* M.p. 205–206 °C. Yield 26%. ^1^H-NMR (300 MHz, DMSO-*d*_6_): δ (ppm) 10.26 (s, 1H, H-1′), 9.12 (d, 2H, *J* = 6.2 Hz, H-2,6), 8.87 (d, 1H, *J* = 6.7 Hz, H-3′), 8.64 (d, 1H, *J* = 6.7 Hz, H-4′), 8.51 (d, 1H, *J* = 8.2 Hz, H-8′), 8.44 (s, 1H, -CH=NOH), 8.38 (d, 1H, *J* = 8.2 Hz, H-5′), 8.32–8.21 (m, 3H, H-3,5,7′), 8.12–8.04 (m, 1H, 6′), 4.83 (t, 2H, *J* = 6.7 Hz, N-CH_2_-), 4.70 (t, 2H, *J* = 6.7 Hz, N′-CH_2_-), 2.18–1.94 (m, 4H, N-CH_2_-CH_2_-). ^13^C-NMR (75 MHz, DMSO-*d*_6_): δ (ppm) 150.08, 148.30, 145.02, 136.92, 136.82, 134.85, 131.09, 130.33, 127.19, 127.13, 125.80, 124.99, 59.67, 59.29, 27.03, 26.81. EA: calculated 48.85% C, 4.53% H, 8.99% N; found 48.53% C, 4.75% H, 8.78% N. ESI-MS: *m*/*z* 153.6 [M]^2+^ (calculated [C_19_H_19_N_3_O]^2+^ 153.59).

*4-hydroxyiminomethyl-1,1′-(but-1,4-diyl)-bispyridinium dibromide* (**K153**). M.p. 243–244 °C. Yield 48%. ^1^H-NMR (300 MHz, DMSO-*d*_6_): δ (ppm) 9.18 (d, 2H, *J* = 6.2 Hz, H-2,6), 9.12 (d, 2H, *J* = 6.2 Hz, H-2′,6′), 8.67–8.59 (m, 1H, H-4′), 8.46 (s, 1H, -CH=NOH), 8.25 (d, 2H, *J* = 6.2 Hz, H-3,5), 8.22–8.15 (m, 2H, H-3′,5′), 4.80–4.62 (m, 4H, N-CH_2_-), 1.97 (m, 4H, N-CH_2_-CH_2_-). ^13^C-NMR (75 MHz, DMSO-*d*_6_): δ (ppm) 148.30, 145.53, 145.01, 144.98, 144.75, 128.05, 124.00, 59.63, 59.19, 27.06, 26.95. EA: calculated 43.19% C, 4.59% H, 10.07% N; found 43.29% C, 4.75% H, 10.08% N. ESI-MS: *m*/*z* 128.6 [M/2]^2+^ (calculated [C_15_H_19_N_3_O/2]^2+^ 128.58).

### 3.2. Biochemical Experiments

**Figure 3:**
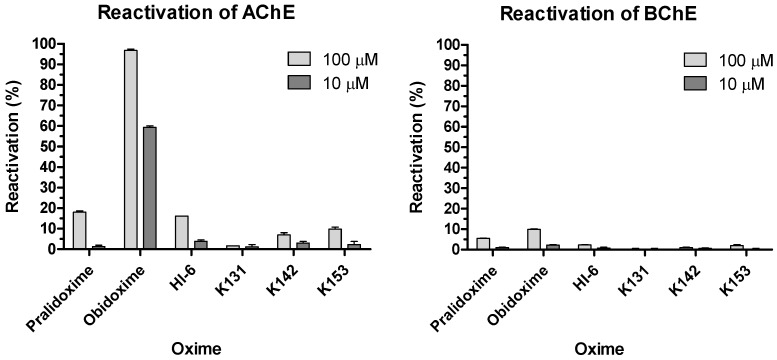
Reactivation of POX-inhibited *Hss*AChE and *Hss*BChE by novel bisquaternary aldoxime reactivators.

All obtained results are summarized in Table 1, and for better visualization, also in Figure 3. According to our results, obidoxime was the most potent compound in reactivation of POX-inhibited *Hss*AChE at both concentrations tested (10 µM and 100 µM), followed by 2-PAM and HI-6. At higher concentration, newly prepared oxime K153 reached a nearly satisfactory reactivation ability (9.8%) for survival of an intoxicated organism. Reactivation abilities of the other two oximes (K131 and K142) were too low for practical use (7.0% and 1.7%, respectively). It seems that smaller pyridinium ring in the molecule (K153) has a more positive influence on the reactivation potency than the larger quinolinium (K131) or isoquinolinium rings (K142).

In the case of *Hss*BChE reactivation, all new compounds were almost ineffective. This result corresponds with the general findings described already, that reactivation of OP-inhibited BChE is very difficult and different from AChE [5,6,23,24]. In this case, only obidoxime at higher concentration achieved reactivation of around 10%. No other oximes (including 2-PAM and HI-6) were able to sufficiently reactivate the POX-inhibited *Hss*BChE.

If obtained results are compared, the new *Hss*AChE reactivators are not better than the clinically used ones, so their further investigation could be not recommended. Due to this, novel structurally different oximes derived from clinically used ones (especially from obidoxime) should be designed and tested for their reactivation potency against POX. When considering *Hss*BChE reactivation, none of the tested oximes could be recommended for further testing.

### 3.3. Docking Studies

The Root Mean Square Deviation (RMSD) obtained for the best redocked structures of HI-6 and BP4M over the crystallographic structures of *Hss*AChE and *Hss*BChE were 1.18 and 1.30 Å, respectively. Considering literature reports that a RMSD value under 2.0 Å is considered acceptable [25], these results validate the docking protocol used for both enzymes.

According to Chemicalize [19] results, 2-PAM and HI-6 present their oxime groups mostly ionized under pH 7.4, with 99.74% and 97.08% of the species, respectively, under this form, while 93.45% of the species of obidoxime and 97.60% of the species of each one of the new oximes present their oxime groups in neutral form under the same pH. Accordingly, the corresponding most abundant forms of each oxime were the ones chosen for the docking studies.

**Figure 4:**
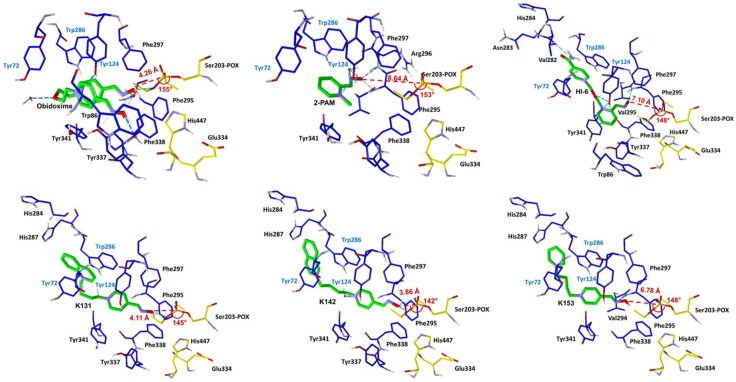
Best poses of the oximes inside the complex *Hss*AChE/POX. Distances P_OP_ − O_Ser203_ and angles O_ox_ − P_OP_ − O_Ser203_ are shown in red.

**Figure 5:**
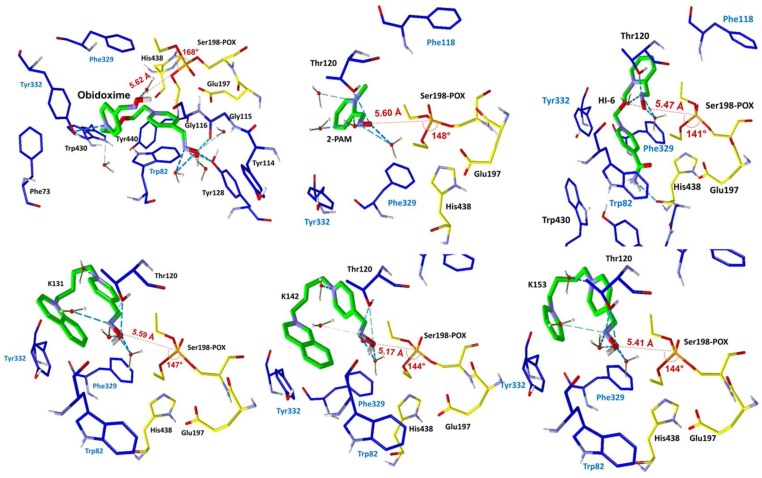
Best poses of the oximes inside the complex *Hss*BChE/POX. Distances P_OP_ − O_Ser203_ and angles O_ox_ − P_OP_ − O_Ser203_ are shown in red.

**Figure 6:**
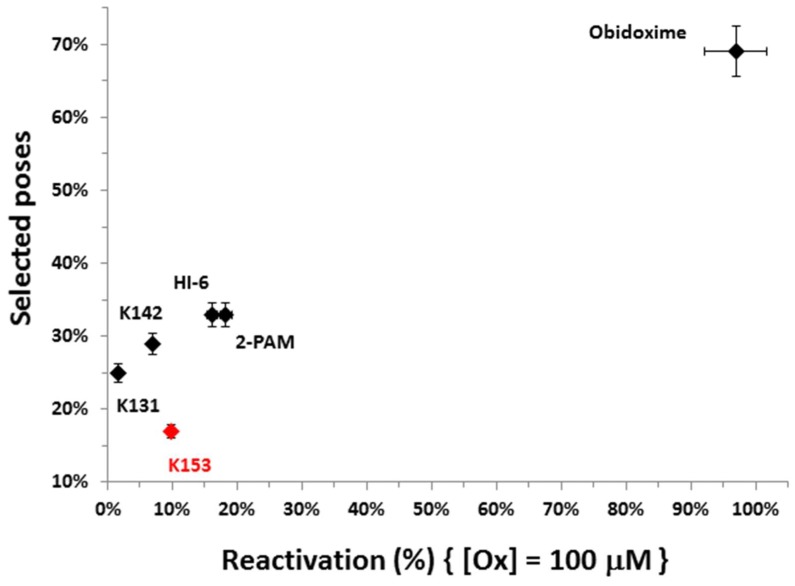
Correlation between the percentage of poses at the near attack conformation (NAC) and the percentage of reactivation for the oximes (Ox) for the complex *Hss*AChE/POX.

The ChE/OP/oxime interaction energies (intermolecular and H-bonds) were calculated in order to elucidate the binding modes of the oximes with the complexes *Hss*AChE/POX and *Hss*BChE/POX and the possible molecular factors responsible for reactivation. Table 2 and Table 3 list the residues interacting with each oxime, the energy values of intermolecular interactions and H-bonds, and the NAC data (d_OP_ and θ_OPO_) observed for the best poses of the oximes (Figure 4 and Figure 5), inside the complexes *Hss*AChE/POX and *Hss*BChE/POX, respectively. These tables show that the best poses of the new oximes (except for 2-PAM) presented similar energy values and also, basically interacted with the same residues as the commercial ones in both systems. As 2-PAM is relatively small compared to the other oximes, it was already expected that it would not be able to establish the same number of stabilizing interactions. Based only on the best pose for each oxime, the results are quite qualitative and reflect only the fact that all oximes have affinities for the enzymes. However, when the discussion moves to the correlations between the percentage of poses at the NAC conditions and the percentage of reactivation, it is possible to observe for the complex *Hss*AChE/POX, in the plot of Figure 6, that the theoretical results fit quite well to the experimental results shown in Figure 3, with the most active reactivator (obidoxime) showing the higher percentage of poses at NAC, followed by 2-PAM and HI-6 (also commercial), and finally the new oximes (worse results). Analyzing the poses returned for the new oximes, it was possible to observe that most poses at the NAC for the complex *Hss*AChE/POX had their pyridinium or quinolinium rings trapped among residues Tyr72, Tyr124, and Trp286 from the peripheral anionic site. We believe that this “trapping”, despite helping to position the oximes at the NAC, contributes to holding these molecules in that position through hydrophobic interactions, then avoiding the approach to attack the OP and start the reactivation reaction. Among the new oximes, the best result of percentage of reactivation was for K153, that possess only one pyridinium ring is smaller than the quinolinium/isoquinolinium moiety of K131 and K142, and therefore, presenting weaker hydrophobic interactions with the residues of the peripheral anionic site. These weaker interactions are also reflected in the higher value of energy shown in Table 2 for this oxime in comparison with K131 and K142. The commercial oximes, on the other hand, presented fewer poses with fewer trapping interactions with residues of the peripheral anionic site, being freer to move and start the reactivation reaction. Also, in our view, the outstanding results presented by obidoxime could be explained by the fact that this reactivator brings oxime groups on both sides, and consequently, doubles the chances of achieving poses at the NAC.

For the complex *Hss*BChE/POX, it was not possible to correlate the percentage of poses at NAC with the percentage of reactivation. In fact, this was also an expected result, because none of the oximes were able to satisfactorily reactivate (more than 10%) the complex *Hss*BChE/POX, as shown in Figure 3. We believe that this happens because the cavity of *Hss*BChE is larger than *Hss*AChE and does not present residues to help molecules, such as the oximes under study here, to adopt the NAC. In our view, this higher difficulty of achieving a stable NAC inside *Hss*BChE could explain the poor percentage of reactivation observed for all oximes with the complex *Hss*BChE/POX. This could be a possible reason why AChE reactivators usually do not work well with BChE.

In order to improve the scaffold of the new oximes studied here and for designing more effective ones, we believe that increasing the bulkiness of their hydrophobic moieties by adding more aromatic rings would help to explore more interactions with the residues of the hydrophobic pockets of both *Hss*AChE and *Hss*BChE. This would contribute to a better stabilization of these oximes inside the enzymes. For the oximes meant to reactivate *Hss*BChE, even bigger hydrophobic moieties should be considered due to the larger cavity present in this enzyme. Also, we believe that increasing the size of the spacer from the current four C atoms to five or six C atoms would introduce more degrees of freedom to the oxime moieties, allowing them to achieve more poses at the NAC, while their hydrophobic moieties are trapped in the hydrophobic pockets. Furthermore, adding heteroatoms, such as O and N, to the spacer could help to improve the water solubility of the oximes, improving their drug abilities.

## 4. Conclusions

We reported the synthesis and in vitro evaluation of three novel bisquaternary aldoximes as potential reactivators for *Hss*AChE and *Hss*BChE inhibited by the pesticide POX, which failed to reactivate these two cholinesterases, with results slightly better for the complex *Hss*AChE/POX. The commercial oximes 2-PAM, obidoxime, and HI-6 were also evaluated and presented good values of percentage of reactivation for the complex *Hss*AChE/POX, with an outstanding difference towards obidoxime. Further docking studies showed a very good correlation between the values of percentage of reactivation and the percentage of poses obtained for the oximes at the NAC for the complex *Hss*AChE/POX, suggesting that the more poses the oxime presents at the NAC, the better its capacity of reactivating the complex. It was also observed that for the new oximes, most poses at the NAC were trapped between the residues Tyr72, Tyr124, and Trp286 at the peripheral anionic site of the narrow gorge of *Hss*AChE. This suggests that the peripheral anionic site, despite playing an important role in helping the oximes to achieve the NAC, can also trap them, avoiding the movement forward to start the reactivation reaction. This argument is reinforced by the fact that this correlation between the values of the percentage of reactivation and the percentage of poses was not observed for the complex *Hss*BChE/POX. We believe that this is due to the fact that *Hss*BChE has a larger cavity, with no peripheral site able to help the oximes to achieve the NAC. This is also a possible explanation for the worse values of percentage of reactivation usually observed for AChE reactivators when tested against BChE. In our view, different scaffolds should be tested, mainly in the case of reactivation of BChE, in order to improve interactions with the peripheral sites. Also, larger spacers should be considered in order to facilitate the adoption of the NAC conformations, due to the trapping of the hydrophobic moieties of the oximes in the peripheral sites.

## Figures and Tables

**Table 1:** Potency of tested oximes to reactivate POX-inhibited erythrocyte *Hss*AChE and plasma *Hss*BChE at concentrations 100 µM and 10 µM. (%, mean value of three independent determinations; time of reactivation by *Hss*AChE reactivators = 10 min; pH 7.4; temperature 25 °C).

Reactivator	Reactivation (%)							
	*Hss*AChE				*Hss*BChE			
	100 µM		10 µM		100 µM		10 µM	
	Mean	SD	Mean	SD	Mean	SD	Mean	SD
pralidoxime	18.2	0.7	1.3	0.7	5.5	0.1	1.0	0.2
obidoxime	96.9	0.7	59.4	0.7	9.9	0.3	2.2	0.3
HI-6	16.1	0.0	3.9	0.7	2.3	0.2	0.8	0.4
K131	1.7	0.0	1.1	1.1	0.3	0.3	0.3	0.3
K142	7.0	1.0	3.0	0.8	1.1	0.2	0.6	0.3
K153	9.8	1.0	2.3	1.5	2.0	0.5	0.3	0.3

**Table 2:** Docking results for the complex *Hss*AChE/paraoxon (POX).

Oxime	The Best Pose (↓d_OP_*/↑θ_OPO_*)					
	d_OP_ (Å)	Angle _OPO_	Energy of Interaction (kcal/mol)	Energy of H-bond (kcal/mol)	Interactions: H-bond	Interactions: Hydrophobic (π–π)
pralidoxime	8.644	153.35°	−73.444	−5.153	Tyr124, Val294/Phe295, Phe295/Arg296	Tyr72, Tyr124, Trp286,Phe295, Phe297, Tyr337, Phe338, Tyr341
obidoxime	4.260	155.00°	−105.013	−4.941	Tyr124, Tyr337 Ser203–POX	Tyr72, Trp86, Tyr124, Trp286, Phe295, Phe297, Tyr337, Phe338, Tyr341, His447
HI-6	7.102	147.64°	−135.261	−6.397	Tyr124, Val294/Phe295, Val282/Asn283	Tyr72, Trp86, Tyr124, His284, Trp286, Phe295, Phe297, Tyr337, Phe338, Tyr341
K131	4.111	144.47°	−136.854	−2.500	Ser203–POX	Tyr72, Tyr124, His284, Trp286, His287, Phe295, Phe297, Tyr337, Phe338, Tyr341, His447
K142	3.864	141.70°	−129.978	−3.586	Tyr72,Ser203–POX	Tyr72, Tyr124, His284, Trp286, His287, Phe295, Phe297, Tyr337, Phe338, Tyr341
K153	6.777	147.81°	−119.168	−2.445	Val294/Phe295	Tyr72, Tyr124, His284, Trp286, His287, Phe295, Phe297, Phe338, Tyr341

* d_OP_ = distance P_OP_ − O_Ser203_; ** θ_OPO_ = angle O_ox_ − P_OP_ − O_Ser203_.

**Table 3:** Docking results for the complex *Hss*BChE/POX.

Oxime	The Best Pose (↓d_OP_/↑θ_OPO_)					
	d_OP_ (Å)	θ_OPO_	Energy of Interaction (kcal/mol)	Energy of H-bond (kcal/mol)	Interactions: H-bond	Interactions: Hydrophobic (π–π)
pralidoxime	5.600	148.21°	−57.770	−2.370	Thr120	Phe118, Phe329, Tyr332
obidoxime	5.623	168.15°	−135.823	−9.600	Gly115/Gly116, Tyr128, Tyr332	Phe73, Trp82, Tyr114, Tyr128, Phe329, Tyr332, Trp430, Tyr440, His438
HI-6	5.469	140.81°	−126.712	−4.851	Thr120, His438/Gly439	Trp82, Phe118, Phe329, Tyr332, Trp430, Tyr440, His438
K131	5.587	146.97°	−118.023	−2.859	Thr120	Trp82, Phe329, Tyr332, His438
K142	5.172	144.04°	−115.435	−2.431	Thr120	Trp82, Phe118, Phe329, Tyr332, His438
K153	5.414	133.13°	−109.310	−3.860	Thr120	Trp82, Phe118, Phe329, Tyr332
